# Conditional diagnostic accuracy according to inflammation status and age for diagnosing tuberculous effusion

**DOI:** 10.1186/s12890-023-02700-4

**Published:** 2023-10-20

**Authors:** Da Som Jeon, Sung-Hoon Kim, Jang Ho Lee, Chang-Min Choi, Hyung Jun Park

**Affiliations:** 1 Division of Pulmonology and Critical Care Medicine, Department of Internal Medicine, Nowon Eulji Medical Center, University of Eulji, Seoul, South Korea; 2 Department of Anesthesiology and Pain Medicine, Asan Medical Center, Ulsan College of Medicine, Seoul, South Korea; 3 Division of Pulmonary and Critical Care Medicine, Department of Internal Medicine, Asan Medical Center, University of Ulsan College of Medicine, Seoul, South Korea; 4 Department of Oncology, Asan Medical Center, Ulsan College of Medicine, Seoul, South Korea; 5 Division of Pulmonology, Department of Internal Medicine, Gumdan Top General Hospital, Incheon, South Korea

**Keywords:** Tuberculosis, Conditional probability, Pleural Disease, Adenosine deaminase

## Abstract

**Background:**

Tuberculous effusion varies from lymphocyte-dominant to neutrophilic effusion according to inflammation status. The criteria of adenosine deaminase (ADA) and lymphocyte/neutrophil (L/N) ratio have yet not been evaluated across different disease conditions.

**Methods:**

Patients who conducted pleural fluid analysis from 2009 to 2019 at Asan Medical Center were included. Criteria (ADA of 50 and L/N ratio of 0.75) were evaluated by quantile subgroups according to age, C-reactive protein (CRP), white blood cell (WBC), and lactate dehydrogenase (LD) by the Monte Carlo simulation method to diagnose tuberculosis. The model for the ADA and L/N ratio was evaluated by AUROC.

**Results:**

Among the 2,918 reviewed cases, 2034 were included with 229 (11.26%) tuberculosis cases. The mean baseline ADA AUROC was 0.88 across all patients. Increased CRP and WBC showed high proportions of neutrophilic tuberculous effusion, with low sensitivity of approximately 45% and 33% in the fifth WBC and CRP groups, respectively. The AUROC of the models decreased with the increase in WBC and CRP groups (ADA model: 0.69 [the top quantile WBC group], 0.74 [the top quantile CRP group]). The AUROC of the models did not show a trend according to the increase in LD and age.

**Conclusion:**

Inflammatory status affects the diagnostic metrics for tuberculous effusion due to the progression of tuberculous effusion. Clinicians should consider the low accuracy of tuberculous effusion criteria in high-inflammatory conditions when diagnosing tuberculosis.

**Supplementary Information:**

The online version contains supplementary material available at 10.1186/s12890-023-02700-4.

## Introduction

Tuberculous pleural effusion is the chronic accumulation of inflammatory fluid in the pleural space caused by the *Mycobacterium tuberculosis* infection of the pleura [1]. A definitive diagnosis is difficult because of the low sensitivity of bacterial culture methods (< 10%: acid-fast bacilli of pleural fluid, 20–30%: *M. tuberculosis* culture of the fluid) [2]. In obscure cases, closed needle pleural biopsy is conducted; however, this invasive method yields only a confirmation of 60–80% for tuberculosis pleurisy [3]. To avoid this invasive technique and facilitate diagnosis, pleural fluid analysis with adenosine deaminase (ADA) and lymphocyte-dominant features have been used. A pleural fluid ADA of 50 U/L cut-off has shown a diagnostic sensitivity of 95% and 89% specificity [3, 4]. Furthermore, a study including pleural effusion analysis as a case-control design reported that lymphocyte and ADA criteria had an AUROC of 0.974 and 58% and 99% sensitivity and specificity, respectively [5–7].

In a previous study, the deep learning model classified the etiology of pleural effusion based on laboratory results and showed the class probabilities [8]. The visualization map revealed that several tuberculosis pleurisy patients were misclassified as bacterial infections. Pleural effusion caused by a bacterial infection was thought to have a low ADA and a predominance of neutrophils, even though previous research demonstrated similarities between bacterial infection and tuberculous effusion in some cases.

Additionally, the diagnostic metrics differed between the ADA [9] and lymphocyte [4] criteria across several meta-analyses. These differences may be attributed to the random sampling deviation across the studies, but several factors concerning patient characteristics could explain the varying diagnostic performance. For example, patients in the older age group had a lower ADA than those in the younger age group [2]. Moreover, the pleural fluid laboratory data may vary between culture-positive or loculated tuberculous effusion, which is more neutrophilic and less lymphocytic in pleural fluid [10]. Given that neutrophilic tuberculous effusion is associated with severe inflammation, serum biomarkers representing inflammation such as lactate dehydrogenase (LD) [11], C-reactive protein (CRP), and white blood cells (WBCs) have been associated with the different stages of tuberculosis [12]. Although the frequency of neutrophilic tuberculous pleurisy is low [11], inflammatory conditions represented by high inflammatory serum biomarkers may increase the risk of misdiagnosis by using previous ADA and lymphocyte criteria. Nevertheless, the difference in diagnostic metrics according to inflammatory status and age has not been well evaluated in the diagnosis of tuberculous effusion.

This study evaluated the ADA and lymphocyte-based criteria for the diagnosis of tuberculous effusion and the different metrics according to age and inflammatory levels in an intermediate tuberculosis burden country, South Korea.

## Methods

### Clinical data

We retrospectively extracted the medical records of patients who underwent pleural effusion cell analysis from 2009 to 2019 at Asan Medical Center (Seoul, South Korea). Patient data were extracted from the in-house system and indexed by de-identifying encrypted patient identification numbers to maintain confidentiality [13, 14]. Laboratory data were extracted if their acquisition date was within 2 weeks of pleural cell count, including the following: blood chemistry, complete blood cell count, pleural fluid cell count, and pleural fluid chemistry. In cases with multiple laboratory results, the latest set before pleural fluid analysis was included for analysis. Patient selection from whole cases was randomly assigned to minimize the selection bias affecting the diagnostic metrics, and selected cases underwent chart review to identify the aetiology of effusion. The final diagnosis of pleural effusion was confirmed through manual chart review by two independent clinicians (DSJ, HJP). Patients meeting the following criteria were excluded: (1) multiple causes as judged by two clinicians, (2) no clear aetiology of the pleural effusion, or (3) pleural fluid cell analysis conducted for post-treatment follow-up only, not at initial diagnosis.

This study was approved by the ethics committee of Asan Medical Center and conducted per the Declaration of Helsinki. The requirement for informed consent was waived by the ethics committee of Asan Medical Center (approval number 2022 − 0455), given the retrospective nature of the study.

### Definition of tuberculous effusion and neutrophil-dominant tuberculous effusion

Definitive tuberculous effusion was defined when *Mycobacterium tuberculosis* was cultured from the pleural fluid. As fewer than 50% of cases were culture-positive [15], “clinical tuberculous effusion” was defined by the improvement of pleural effusion on chest X-ray after anti-tuberculosis treatment plus one of the following conditions: (i) pleural effusion with a pulmonary tuberculosis lesion, (ii) pathologic findings of granuloma in the pleural biopsy, or (iii) tuberculosis suspected by imaging tests and tuberculosis drugs initiated according to the judgment of the treating clinician. The neutrophil-dominant tuberculous effusion was defined by neutrophils comprising more than 50% of the nucleated cells in the pleural fluid cell analysis [12].

### Non-tuberculous pleural effusion

The alternative pleural effusion aetiology was used as a true negative label. The other aetiologies were categorized as “bacterial infection”, “malignancy”, “volume overload”, and “miscellaneous.” Bacterial infection was defined when bacterial culture was positive for pleural effusion or improved effusion after using adequate antibiotics, excluding other causes. Malignant effusion was defined as a malignant cell identified in the pleural fluid, or if pleural metastasis was suspected in the imaging study without evidence of other causes. Volume overload was defined when definitive causes such as huge ascites or heart failure were identified, and the effusion was resolved through volume control.

### Subgroups by inflammatory markers

The inflammatory laboratory markers were selected from the routine laboratory check-up list in our cohort dataset. The blood inflammatory data were used to define the prior probability through inflammatory biomarkers before the ADA and L/N criteria. The WBCs, CRP, and LD were selected to represent the systemic inflammatory markers and used to define the subgroups. The dataset was divided equally into five quantiles (20% each) to evaluate the trend of diagnostic metrics including age and levels of inflammatory markers. Among the quantile groups, the distribution of neutrophil-dominant tuberculous and ADA was evaluated to identify the cause of the variation in the diagnostic results according to the level of inflammation and age.

### The cut-off of ADA and lymphocytes/neutrophil ratio for diagnosis of tuberculous effusion

A previous meta-analysis demonstrated the various diagnostic cut-offs of ADA [9] and lymphocyte/neutrophil (L/N) ratio [16]. In this analysis, the major cut-off was defined as an ADA of 50 U/L and L/N ratio of 0.75 [3], which was used to evaluate the diagnostic metrics (sensitivity and specificity) in each quantile group. The lymphocyte ratio was calculated by the proportion of lymphocytes among the whole nucleated cells in the pleural fluid. When the neutrophil and lymphocyte numbers were zero, the ratio was zero.

### Statistical analysis

The proportion of neutrophil-dominant tuberculous effusion in each quantile group and the aetiologies of effusion are presented in a bar plot. The group difference in the proportions was analyzed using the chi-square test. The distribution of ADA level across each quantile group according to tuberculous and non-tuberculous effusion is described in a box plot. A one-way ANOVA test was conducted to compare the ADA distribution across the five groups. The diagnostic metrics (sensitivity, specificity, and AUROC) were calculated based on true tuberculosis and others, including malignant effusion, bacterial infections, volume overload, and miscellaneous. Sensitivity and specificity were simulated through a random selection of cases, allowing replacement to have a dataset A of 1000 cases. Sensitivity and specificity were calculated among dataset A, representing each quantile group. Cases with missing data were omitted from the simulation analysis. The dataset was split randomly at 3:7 for training and testing to calculate the AUROC according to the ADA and L/N ratio. A generalized linear model using binomial function was used for model 1 using only the ADA and model 2 using both the ADA and L/N ratio. This process was repeated 1000 times per simulation, and the diagnostic metrics were presented as the median, interquartile box, and 1.5 times standard deviation in a box plot. Paired *t*-tests were performed to compare models 1 and 2 in every simulated five-quantile group. The statistical analysis was performed using R software version 4.1.2 (R Foundation for Statistical Computing, Vienna, Austria).

## Results

### Data description and baseline diagnostic performance of ADA and L/N ratio

**Fig. 1:**
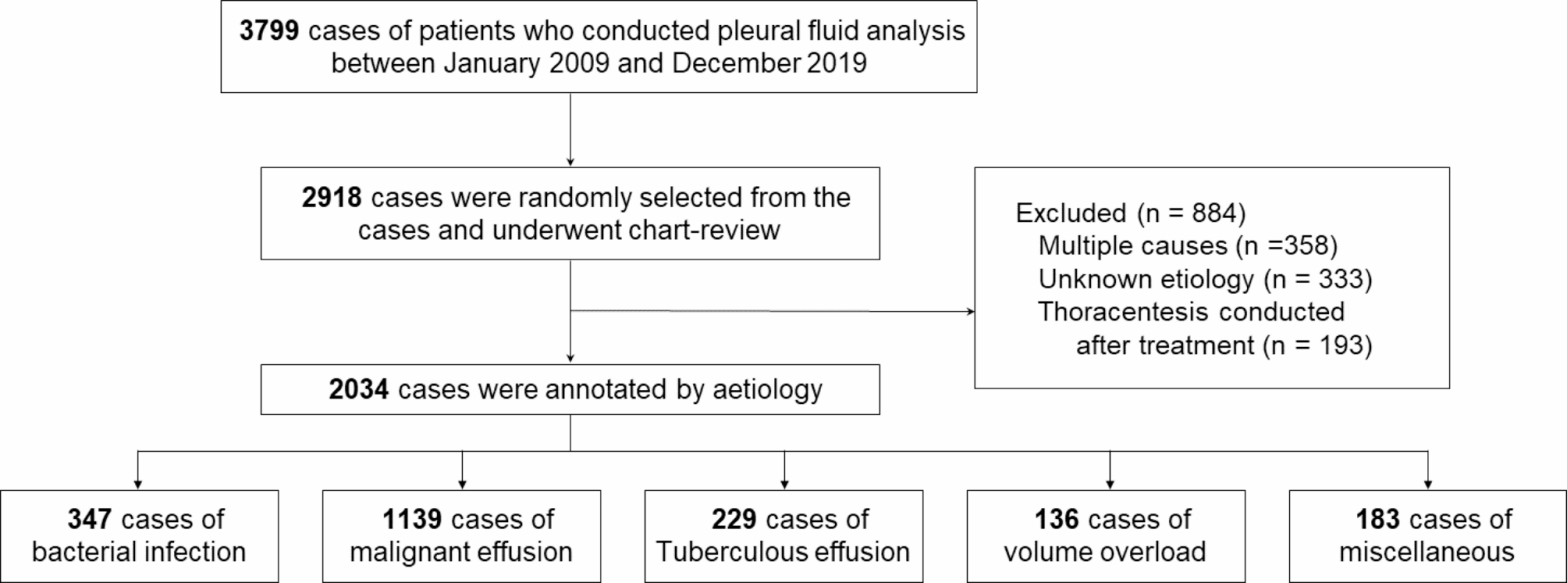
Flow chart of patient case inclusion. From the data warehouse, patients were randomly selected and a chart review was performed to identify the aetiology of pleural effusions. After excluding 884 patients for the above reasons, 2034 cases were analyzed

In our clinical data warehouse, a total of 3,799 pleural effusion analyses were conducted between 2009 and 2019. Among these, 2,918 cases were reviewed by manual chart review, through which 884 cases were excluded according to the aforementioned criteria. Finally, 2,034 patients were included in this study, with a total of 229 tuberculosis cases (Fig. 1). The baseline characteristics of the demographic and laboratory values have been described according to the aetiology of pleural effusion (Table 1). Lactate dehydrogenase data were missing in 308 cases (15.1%), and CRP data were missing in 233 cases (11.4%). The remaining laboratory values had a mean missing data percentage of 2.76%. At an ADA cut-off of 50 U/L and L/N ratio of 0.75, the sensitivity and specificity were 72.49% and 95.79%, respectively. The AUROC of models 1(ADA alone) and 2 (ADA + L/N ratio) were 0.88 and 0.77, respectively.

**Table 1:** Baseline characteristics according to the aetiology of pleural effusion

	Bacterial (N = 347)	Malignancy (N = 1139)	Tuberculosis (N = 229)	Volume overload (N = 136)	Miscellaneous (N = 183)	p-value
**Height (cm)**	164.9 ± 10.0	161.5 ± 9.6	161.4 ± 17.2	161.0 ± 9.0	164.5 ± 6.5	< 0.001
**Body weight (kg)**	61.0 ± 12.2	59.9 ± 10.8	60.5 ± 11.2	57.5 ± 11.9	60.0 ± 10.1	0.054
**Age (years)**	66.1 ± 14.4	65.9 ± 12.3	61.3 ± 18.0	74.0 ± 11.9	63.0 ± 14.6	< 0.001
**Sex**						< 0.001
**Male**	269 (77.5%)	622 (54.6%)	129 (56.3%)	70 (51.5%)	136 (74.3%)	
**Female**	68 (19.6%)	448 (39.3%)	63 (27.5%)	53 (39.0%)	38 (20.8%)	
**Laboratory findings (**CBC**)**						
**WBC (10** ^**3**^ **/uL)**	12.9 ± 6.8	8.9 ± 4.4	7.1 ± 2.4	8.0 ± 4.5	9.7 ± 16.4	< 0.001
**Haemoglobin (g/dL)**	11.4 ± 2.0	12.6 ± 1.9	12.6 ± 2.0	11.2 ± 2.2	12.3 ± 2.3	< 0.001
**MCV (fl.)**	91.5 ± 6.6	91.6 ± 5.4	90.4 ± 6.3	93.7 ± 6.6	90.8 ± 6.4	< 0.001
**MCH (pg)**	30.2 ± 2.6	30.3 ± 2.1	30.0 ± 2.4	30.7 ± 2.6	30.0 ± 2.6	0.013
**PDW (fl.)**	10.7 ± 2.5	10.6 ± 1.9	10.4 ± 2.4	11.7 ± 3.1	10.6 ± 1.7	< 0.001
**Haematocrit (%)**	34.4 ± 5.7	37.9 ± 5.4	37.9 ± 5.4	34.1 ± 6.4	37.3 ± 6.7	< 0.001
**Platelet (10** ^**3**^ **/uL)**	305.3 ± 136.1	295.2 ± 108.3	302.6 ± 103.8	197.4 ± 103.8	265.0 ± 110.3	< 0.001
**Laboratory findings (Chemical)**						
**ALT** ^**1**^	28.5 ± 28.2	23.4 ± 38.0	25.0 ± 31.6	36.6 ± 81.8	26.5 ± 48.1	0.006
**AST** ^**1**^	32.1 ± 26.3	29.2 ± 51.4	28.1 ± 22.8	44.9 ± 58.1	30.2 ± 38.1	0.003
**Alkaline phosphatase** ^**1**^	117.6 ± 74.3	109.5 ± 160.0	90.4 ± 50.4	103.2 ± 68.8	109.2 ± 220.7	0.264
**Total bilirubin (mg/dL)**	0.9 ± 1.2	0.6 ± 0.4	0.6 ± 0.3	1.1 ± 1.5	0.6 ± 0.6	< 0.001
**Glucose (mg/dL)**	145.0 ± 62.4	133.5 ± 54.7	126.9 ± 58.6	141.2 ± 71.1	128.7 ± 48.6	0.001
**Albumin (g/dL)**	2.6 ± 0.6	3.1 ± 0.6	3.2 ± 0.5	2.9 ± 0.6	3.1 ± 0.7	< 0.001
**Total protein (g/dL)**	6.3 ± 0.9	6.5 ± 0.7	6.8 ± 0.8	6.3 ± 1.0	6.5 ± 0.9	< 0.001
**Creatinine (mg/dL)**	1.2 ± 1.5	0.9 ± 0.8	0.9 ± 0.5	1.5 ± 1.6	1.1 ± 1.3	< 0.001
**BUN (mg/dL)**	18.3 ± 12.7	15.8 ± 9.4	14.0 ± 8.3	23.3 ± 15.0	16.0 ± 8.8	< 0.001
**LD** ^**1**^	222.8 ± 81.1	309.5 ± 366.5	220.4 ± 78.8	288.8 ± 108.9	240.1 ± 113.0	< 0.001
**CRP (mg/dL)**	14.7 ± 9.8	4.3 ± 5.9	6.3 ± 6.0	3.1 ± 4.9	4.9 ± 6.3	< 0.001
**Procalcitonin (ng/mL)**	3.3 ± 10.3	1.1 ± 5.8	0.3 ± 0.8	1.3 ± 3.2	0.7 ± 3.0	0.002
**Calcium (mg/dL)**	8.4 ± 0.6	8.9 ± 0.7	8.7 ± 0.6	8.5 ± 0.7	8.6 ± 0.7	< 0.001
**Sodium (mmol/L)**	136.2 ± 4.5	137.8 ± 4.2	137.1 ± 3.8	136.9 ± 4.8	137.8 ± 3.7	< 0.001
**Chloride (mmol/L)**	99.9 ± 5.2	100.9 ± 4.5	101.2 ± 4.2	101.1 ± 6.6	101.5 ± 3.9	0.001
**Potassium (mmol/L)**	4.2 ± 0.6	4.3 ± 0.5	4.2 ± 0.4	4.2 ± 0.6	4.3 ± 0.5	0.01
**Phosphorus (mg/dL)**	3.2 ± 0.9	3.5 ± 0.7	3.3 ± 0.6	3.4 ± 0.9	3.4 ± 0.9	< 0.001
**D-dimer (ug/ml)**	4.4 ± 6.2	4.6 ± 6.3	4.2 ± 4.2	4.4 ± 6.7	3.0 ± 4.4	0.324
**BNP (pg/mL)**	251.3 ± 455.4	138.4 ± 318.5	193.8 ± 421.6	845.4 ± 1253.9	222.1 ± 764.5	< 0.001
**Pleural fluid chemical**						
**ADA** ^**1**^	90.7 ± 141.5	28.6 ± 50.9	88.3 ± 54.4	11.8 ± 8.8	34.4 ± 29.1	< 0.001
**LD** ^**1**^	4919.5 ± 10686.8	1074.2 ± 5184.3	727.1 ± 1864.9	149.1 ± 309.0	802.7 ± 1257.3	< 0.001
**Albumin (g/dL)**	2.0 ± 0.8	2.6 ± 0.7	2.7 ± 0.6	1.4 ± 0.6	2.3 ± 0.7	< 0.001
**Total protein (g/dL)**	4.1 ± 1.3	4.4 ± 1.0	4.9 ± 1.0	2.5 ± 1.0	4.3 ± 1.2	< 0.001
**Glucose (mg/dL)**	89.2 ± 80.8	109.9 ± 54.9	101.5 ± 48.8	156.8 ± 63.3	106.6 ± 57.1	< 0.001
**pH**	7.2 ± 0.3	7.3 ± 0.2	7.3 ± 0.2	7.4 ± 0.1	7.3 ± 0.2	< 0.001
**Pleural fluid cell count**						
**Nucleated cells (/uL)**	31.90 ± 85.5	6.20 ± 4.12	6.73 ± 22.40	1.12 ± 2.31	6.10 ± 15.82	< 0.001
**RBC (/uL)**	59.61 ± 261.43	70.82 ± 270.61	48.34 ± 322.84	40.37 ± 282.84	255.62 ± 694.30	< 0.001
**Histiocyte (%)**	14.3 ± 15.8	27.9 ± 19.5	18.2 ± 14.5	44.0 ± 21.4	25.0 ± 20.6	< 0.001
**Neutrophil (%)**	65.2 ± 33.0	18.2 ± 24.4	18.5 ± 23.8	15.4 ± 21.6	30.6 ± 30.3	< 0.001
**Lymphocyte (%)**	19.3 ± 22.8	39.2 ± 25.5	63.3 ± 25.7	37.5 ± 21.7	34.5 ± 28.3	< 0.001
**Eosinophil (%)**	3.9 ± 6.0	5.4 ± 8.0	5.8 ± 9.6	7.5 ± 15.0	17.1 ± 24.7	< 0.001
**Basophil (%)**	1.5 ± 1.1	1.5 ± 1.1	1.5 ± 1.4	1.9 ± 1.8	1.7 ± 1.3	0.473
**Mesothelial cell (%)**	4.5 ± 6.0	4.0 ± 4.7	2.8 ± 2.9	3.7 ± 4.5	4.1 ± 5.1	0.087
**Complicated pleural effusion**						< 0.001
**False**	276 (79.5%)	1100 (96.6%)	223 (97.4%)	136 (100.0%)	176 (96.2%)	
**True**	71 (20.5%)	39 (3.4%)	6 (2.6%)	0 (0.0%)	7 (3.8%)	

^1^: the unit is U/L.

### Proportion of neutrophil dominant tuberculous effusions and ADA distribution

**Fig. 2:**
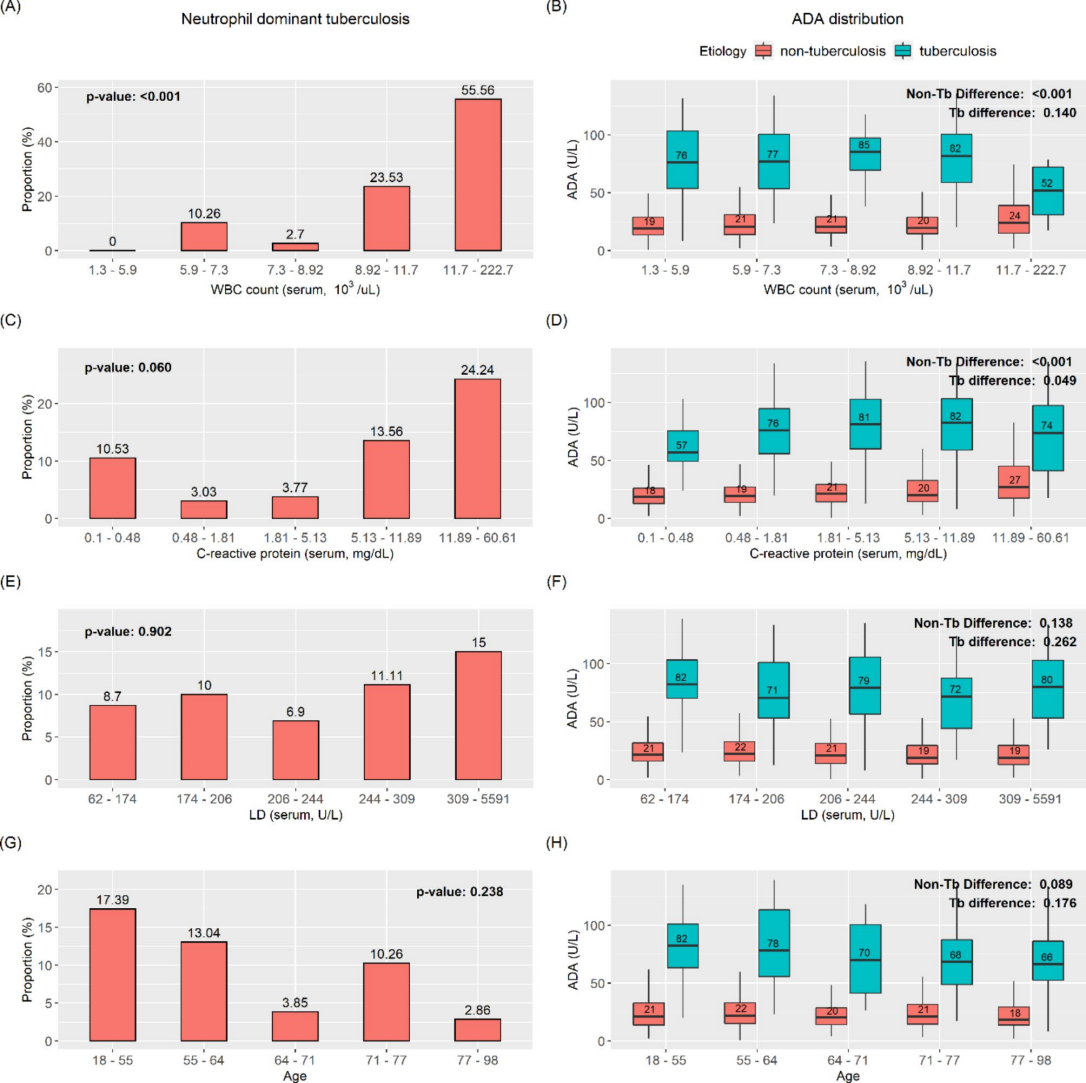
Neutrophil-dominant tuberculous effusion and ADA distribution according to quantile group. (A, C, E, G) The neutrophil-dominant tuberculous effusion (pleural neutrophil more than 50%) among total tuberculosis cases in each quantile group are depicted by bar plots. Higher WBC (A) and CRP (C) were associated with neutrophil-dominant tuberculosis, as well as younger age (B, D, F, H). Box plots depict the ADA distribution according to tuberculosis or non-tuberculosis of each quantile group. For clarity, the outliers are not shown in these box plots. With respect to the non-tuberculous ADA distribution, higher WBC (A) and CRP (C) were associated with increased ADA level and distribution

All data were categorized according to inflammatory markers (WBC, CRP, and LD) and age. The trend of neutrophil-dominant proportion and ADA distribution by aetiology is presented in Fig. 2. Neutrophilic-dominant tuberculous effusion was more frequently observed as levels of WBC and CRP increased (*p*: <0.001, *p*: 0.060, respectively, Fig. 2-A, C). Additionally, the younger age group had a greater proportion of neutrophilic-dominant tuberculous effusion (*p*: 0.238, Fig. 2-G). However, LD was not significantly associated with the proportion of neutrophil-dominant tuberculous effusion (*p*: 0.902).

A higher WBC was associated with an increase in mean ADA and distribution of ADA in non-tuberculous effusion (*p*: <0.001, *p*: 0.140, respectively non-TB and TB, Fig. 2-B), which was similarly observed in the CRP group (*p*: <0.001, *p*: 0.049). The high mean and wider distribution of ADA reduced the diagnostic performance due to the poor discriminative value of ADA in tuberculous and non-tuberculous effusion. Thus, patients with higher WBC and CRP levels are expected to have lower diagnostic metrics. Changes in LD and age did not significantly impact the distribution of ADA (*p*: LD = 0.138 (non-TB), 0.262 (TB); age = 0.089 (non-TB), 0.176 (TB)).

### Simulation analysis of diagnostic metrics according to inflammatory markers and age

**Fig. 3:**
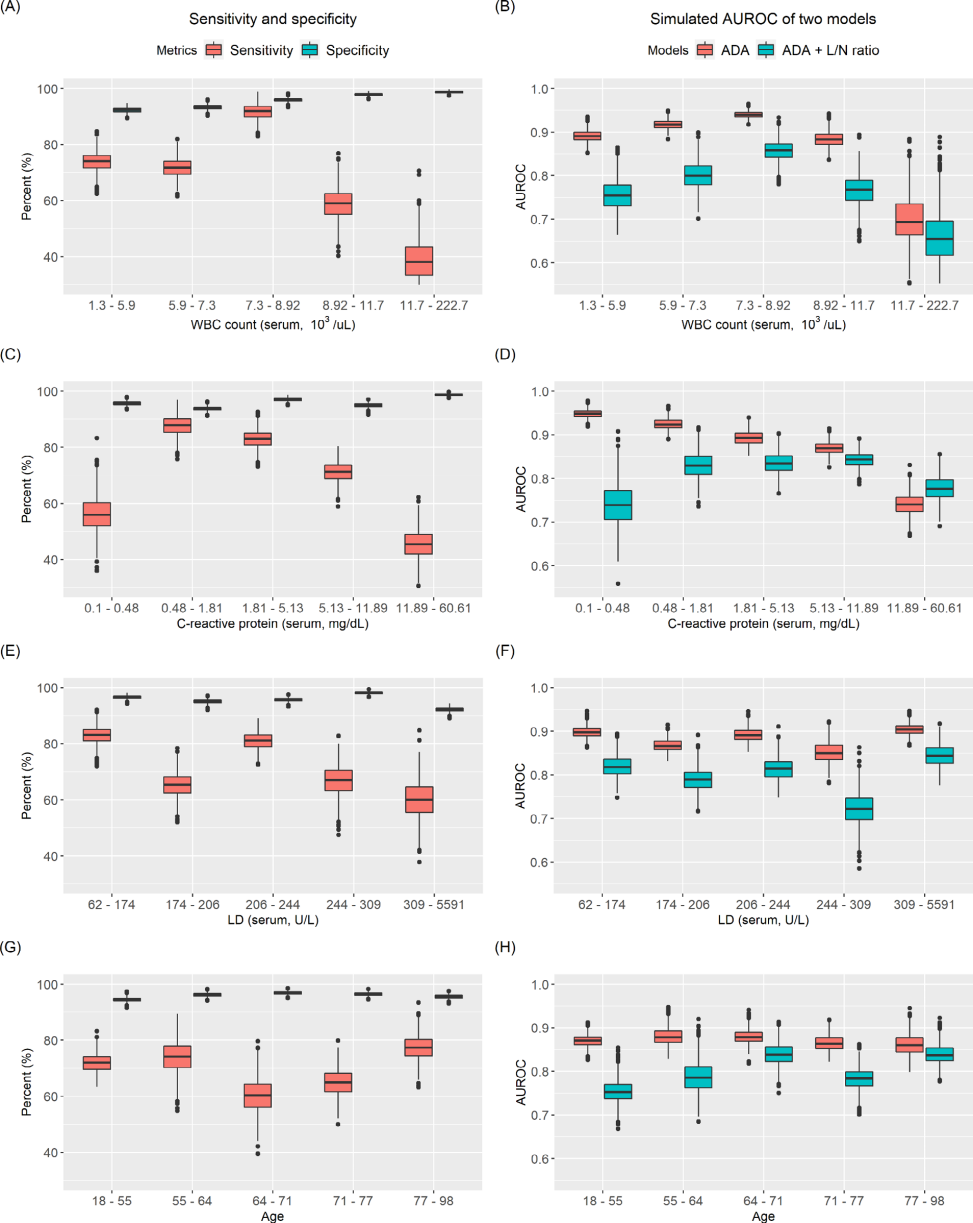
Diagnostic metrics according to quantile groups by inflammatory marker and age. (A, C, E, G) The sensitivity and specificity of the five quantile groups are depicted by box plots. The distribution of the sensitivity and specificity results from the Monte-Carlo simulation of random sampling in each group are displayed. The higher WBC and CRP were associated with low sensitivity (B, D, F, H). The AUROC distribution of each model using ADA (model 1) and ADA with L/N ratio (model 2) are presented as box plots. The higher WBC and CRP were associated with low AUROCs

The diagnostic metrics when using the cut-off of ADA 50 U/L and L/N ratio of 0.75 according to inflammatory markers and age are depicted in Fig. 3. The fourth and fifth WBC groups had a lower sensitivity (median: 59% and 33%, respectively) compared with the other three groups (Fig. 3-A, Table 2). Additionally, the first CRP group (lowest) and fifth group (highest) exhibited low sensitivity (median: 56% and 45%, respectively) (Fig. 3-C). With respect to LD and age, all groups showed a sensitivity of more than 60% and no specific drop in sensitivity by the quantile group (Fig. 3-E, G).

**Table 2:** Summary statistics of sensitivity and specificity according to quantile group

	Quantile 1	Quantile 2	Quantile 3	Quantile 4	Quantile 5
WBC	1.3–5.9	5.9–7.3	7.3–8.92	8.92–11.7	11.7–222.7
Sensitivity	74.1 [71.64‒76.1]	71.73 [69.39‒74.06]	91.95 [89.9‒93.64]	59.01 [55.04‒62.5]	33.33 [26.09‒40]
Specificity	92.29 [91.63‒92.93]	93.32 [92.73‒93.9]	95.88 [95.46‒96.34]	97.9 [97.56‒98.21]	98.76 [98.46‒98.97]
CRP	0.1–0.48	0.48–1.81	1.81–5.13	5.13–11.89	11.89–60.61
Sensitivity	55.97 [51.94‒60.29]	87.91 [85.39‒90.14]	83 [80.95‒85.06]	71.34 [68.82‒73.68]	45.41 [41.74‒48.89]
Specificity	95.7 [95.22‒96.15]	93.79 [93.25‒94.34]	97.1 [96.66‒97.5]	95.06 [94.56‒95.56]	98.79 [98.56‒99.01]
LD	62–174	174–206	206–244	244–309	309–5591
Sensitivity	83.2 [81.05‒85.04]	65.49 [62.5‒68.13]	81.1 [79.07‒83.15]	67.01 [63.28‒70.51]	60 [55.56‒64.52]
Specificity	96.66 [96.23‒97.05]	95.13 [94.63‒95.62]	95.73 [95.27‒96.19]	98.14 [97.84‒98.45]	92.26 [91.69‒92.83]
Age	18–55	55–64	64–71	71–77	77–98
Sensitivity	71.96 [69.64‒74.14]	74.14 [70.18‒77.97]	60.27 [56.05‒64.41]	64.95 [61.61‒68.22]	77.32 [74.49‒80.24]
Specificity	94.52 [94‒95.02]	96.25 [95.8‒96.68]	96.89 [96.47‒97.25]	96.49 [96.08‒96.89]	95.6 [95.13‒96.06]

The statistics are summarised as the median and inter-quantile range.

The quantile groups were evaluated by a logistic model using ADA alone and ADA with L/N ratio according to inflammatory markers and age. The AUROCs of the models for diagnosing tuberculosis are depicted in Fig. 3. Overall, the AUROC of model 1 (ADA alone) outperformed that of model 2 (ADA and L/N ratio) (Fig. 3). The highest WBC group had comparatively low AUROCs of 0.69 (model 1) and 0.65 (model 2) (Fig. 3-B, Table 3). In the CRP group, the higher CRP group had lower AUROC in model 1, and the fifth CRP group had 0.74 in model 1 (Fig. 3-D, Table 3). The AUROC of model 2 in the first and fifth CRP groups also showed a comparatively low performance (median: 0.74 and 0.78, respectively) (median: 0.83‒0.84). In particular, the first CRP group exhibited a much lower performance in model 2 than in model 1 (0.74 vs. 0.95, respectively). The AUROC of model 1 did not differ significantly among the quantile groups in the LD and age quantile groups (Fig. 3-F, H). In model 2, the younger age group (first, second) had a lower AUROC than the older age groups (third to fifth).

**Table 3:** Summary statistics of AUROC according to quantile group by model 1 and model 2

	Quantile 1	Quantile 2	Quantile 3	Quantile 4	Quantile 5
WBC	1.3–5.9	5.9–7.3	7.3–8.92	8.92–11.7	11.7–222.7
Model 1	0.89 [0.88‒0.9]	0.92 [0.91‒0.92]	0.94 [0.93‒0.94]	0.88 [0.87‒0.9]	0.69 [0.66‒0.74]
Model 2	0.75 [0.73‒0.78]	0.8 [0.78‒0.82]	0.86 [0.84‒0.87]	0.77 [0.74‒0.79]	0.65 [0.61‒0.69]
CRP	0.1–0.48	0.48–1.81	1.81–5.13	5.13–11.89	11.89–60.61
Model 1	0.95 [0.94‒0.95]	0.92 [0.92‒0.93]	0.89 [0.88‒0.9]	0.87 [0.86‒0.88]	0.74 [0.72‒0.76]
Model 2	0.74 [0.71‒0.77]	0.83 [0.81‒0.85]	0.83 [0.82‒0.85]	0.84 [0.83‒0.85]	0.78 [0.76‒0.8]
LD	62–174	174–206	206–244	244–309	309–5591
Model 1	0.9 [0.89‒0.91]	0.87 [0.86‒0.88]	0.89 [0.88‒0.9]	0.85 [0.84‒0.87]	0.9 [0.9‒0.91]
Model 2	0.82 [0.8‒0.84]	0.79 [0.77‒0.81]	0.81 [0.8‒0.83]	0.72 [0.7‒0.75]	0.84 [0.83‒0.86]
Age	18–55	55–64	64–71	71–77	77–98
Model 1	0.87 [0.86‒0.88]	0.88 [0.87‒0.89]	0.88 [0.87‒0.89]	0.86 [0.85‒0.88]	0.86 [0.84‒0.88]
Model 2	0.75 [0.74‒0.77]	0.79 [0.76‒0.81]	0.84 [0.82‒0.86]	0.78 [0.77‒0.8]	0.84 [0.82‒0.85]

Model 1 denotes the logistic regression model using ADA to predict tuberculous effusion. Model 2 used ADA and lymphocyte/neutrophil ratio to predict tuberculous effusion. The AUROC is summarised as the mean and inter-quantile values of all simulated sampled data.

## Discussion

In this study, the diagnostic metrics for tuberculosis effusion differed according to inflammatory status. When the WBC was greater than 11.8 × 10^3^ /L or the CRP was higher than 11.9 mg/dL, the AUROCs of model 1 (ADA alone) and model 2 (ADA + L/N ratio) were lower than the other quantile groups, and the sensitivity was lower than 40% and 30%, respectively. High inflammatory conditions had a greater proportion of neutrophil-dominant tuberculous effusion and wider ADA distributions of non-tuberculous effusion, suggesting that the high inflammatory status of tuberculosis could easily be missed by the current ADA and L/N ratio cut-offs. Moreover, the younger age group had a lower AUROC in model 2 (ADA, L/N ratio) in line with the higher rate of neutrophil-dominant tuberculous effusion. However, there was no clear association with age in model 1 (ADA alone).

Tuberculous effusion is believed to be a delayed hypersensitivity reaction to the tuberculous protein [15], while fluid and inflammatory responses become more prominent in line with the increase in mycobacterial burden [10]. Culture-positive tuberculosis effusion tends to have fewer lymphocytes in the pleural fluid and a high level of CRP [17], suggesting that higher inflammatory effusion is more neutrophilic, affecting the diagnostic cut-off of ADA and L/N ratio. This study assumed that serum WBC and CRP could represent the inflammatory status of effusion. As the inflammation progressed, the neutrophilic-dominant tuberculous effusion increased in this study. Moreover, higher levels of inflammation, such as pleural thickening, were associated with increased ADA in non-tuberculous effusion in a previous study [18]. Our study supports the findings that the non-tuberculous effusion exhibits higher ADA distribution as inflammation progresses. Consequently, the diagnostic accuracy of ADA and the L/N ratio decreased at high inflammatory status, with the sensitivity lowering to 30% at an ADA of 50 and L/N ratio of 0.75. To improve sensitivity, we simulated various cut-offs such as an ADA of 35 to 70 and L/N ratio of 0.3 to 0.75. We observed that an L/N ratio of 0.3 increased sensitivity without decreasing specificity below 95% at high inflammatory status (sensitivity; fifth WBC: 59%, fifth CRP 69%) (Supplement Figs. 1–4).

Previous studies have demonstrated a difference in serum LD levels in culture-positive and culture-negative tuberculous effusion [14]. Although LD can be used to assess the inflammatory status of respiratory disease [19], it is also a well-known biomarker for lymphoma severity [20]. In our analysis, a higher LD level was not associated with neutrophil-dominant tuberculous effusion nor the ADA distribution of non-tuberculous effusion. Moreover, the increase in LD was not associated with a higher proportion of bacterial infection but with malignant effusion (Supplement 5). Representation of inflammation through LD was therefore more likely to be associated with non-infectious than infectious causes. Consequently, the discriminative performance was not associated with an increase in LD level.

A previous study reported that ADA decreased with increasing age in patients with tuberculous effusion [2], which may impact the diagnostic metrics. This study also noted a negative correlation between age and ADA level in tuberculosis (Supplement Fig. 6), which could affect the sensitivity of ADA-based criteria for tuberculous effusion. However, we also demonstrated that the AUROC of model 1 (ADA alone) did not significantly drop in the older age group, which could be attributed to the division of the groups by quantile age of the included patients, which was skewed to older age. Thus, the younger group included patients aged up to 55 years, which could devalue the observed trend in AUROC of model 1 according to age. Additionally, the younger age group exhibited a greater proportion of neutrophilic tuberculous effusion, the AUROC of model 2 (ADA + L/N ratio) was lower in the younger age group. However, in previous studies, the younger age was not associated with progressed tuberculous effusion in culture-positive [11] or loculated effusion [17]. These findings should be further evaluated in future studies.

There are some limitations to this study. First, despite including the details of all pleural fluid analyses conducted over the past 10 years, this only totaled 229 cases, which may not be enough to determine the subtype of tuberculous effusion. However, to our knowledge, this is the largest study of pleural effusion cases comparing the sublevel of patients included by random sampling methods. This method should be subject to the lowest inclusion bias, which could evaluate the conditional probability defined by the laboratory data. Second, this study did not consider the radiologic findings and clinical symptoms that could affect the conditional status. Our data collection method was auto-extraction from the data warehouse, so this type of data collection and annotation was not available. Third, in general, cases of microbiologically confirmed tuberculous pleural effusion are not common [21], Therefore, some argue that there may be a misdiagnosis of tuberculous effusion in this study. We defined tuberculous pleural effusion if there was suspicion based on clinical, laboratory, and radiological evidence, and then observed clinical improvement while maintaining appropriate anti-tuberculosis treatment for at least 3 months. Therefore, we believe the possibility of misdiagnosis is very low.

## Conclusions

Inflammatory status defined by WBC and CRP affects the diagnostic performance of ADA and lymphocyte-to-neutrophil ratio criteria for tuberculous effusion. Clinicians should consider the false-negative cases of tuberculous effusion in high-inflammatory conditions and conditional differences in diagnostic performance.

### Electronic supplementary material

Below is the link to the electronic supplementary material.

Supplementary Material 1

## Data Availability

The datasets generated and/or analyzed during the current study are not publicly available due to patients’ personal information but are available from the corresponding author upon reasonable request.
