# Correction: Conditional diagnostic accuracy according to inflammation status and age for diagnosing tuberculous effusion

**DOI:** 10.1186/s12890-023-02769-x

**Published:** 2023-11-23

**Authors:** Da Som Jeon, Sung-Hoon Kim, Jang Ho Lee, Chang-Min Choi, Hyung Jun Park

**Affiliations:** 1 Division of Pulmonology and Critical Care Medicine, Department of Internal Medicine, Nowon Eulji Medical Center, University of Eulji, Seoul, South Korea; 2 Department of Anesthesiology and Pain Medicine, Asan Medical Center, Ulsan College of Medicine, Seoul, South Korea; 3 Division of Pulmonary and Critical Care Medicine, Department of Internal Medicine, Asan Medical Center, University of Ulsan College of Medicine, Seoul, South Korea; 4 Department of Oncology, Asan Medical Center, Ulsan College of Medicine, Seoul, South Korea; 5 Division of Pulmonology, Department of Internal Medicine, Gumdan Top General Hospital, Incheon, South Korea

**Correction: BMC Pulmonary Medicine 23, 400 (2023)**

**https://doi.org/10.1186/s12890-023-02700-4**

Following publication of the original article [1], it came to the authors' attention that the second author, Sung-Hoon Kim, had been misspelled as Seoung Hoon Kim. The name has been corrected in the original article and the corrected name may be seen in the author list of this erratum. The authors thank you for reading and apologize for any inconvenience caused.
