# Supplementary material for: Conditional diagnostic accuracy according to inflammation status and age for diagnosing tuberculous effusion
Source: BMC Pulm Med. 2023 Oct 20;23:400. doi: 10.1186/s12890-023-02700-4 (PMC10589957; doi:10.1186/s12890-023-02700-4)
Supplement: Supplementary file 1 — Supplementary Material 1 [file 12890_2023_2700_MOESM1_ESM.docx]

Supplement

**Conditional diagnostic accuracy according to inflammation status and age for diagnosing tuberculous effusion**

Supplement Figure 1. Diagnostic metrics according to various cut-off criteria for tuberculous effusion by WBC group.


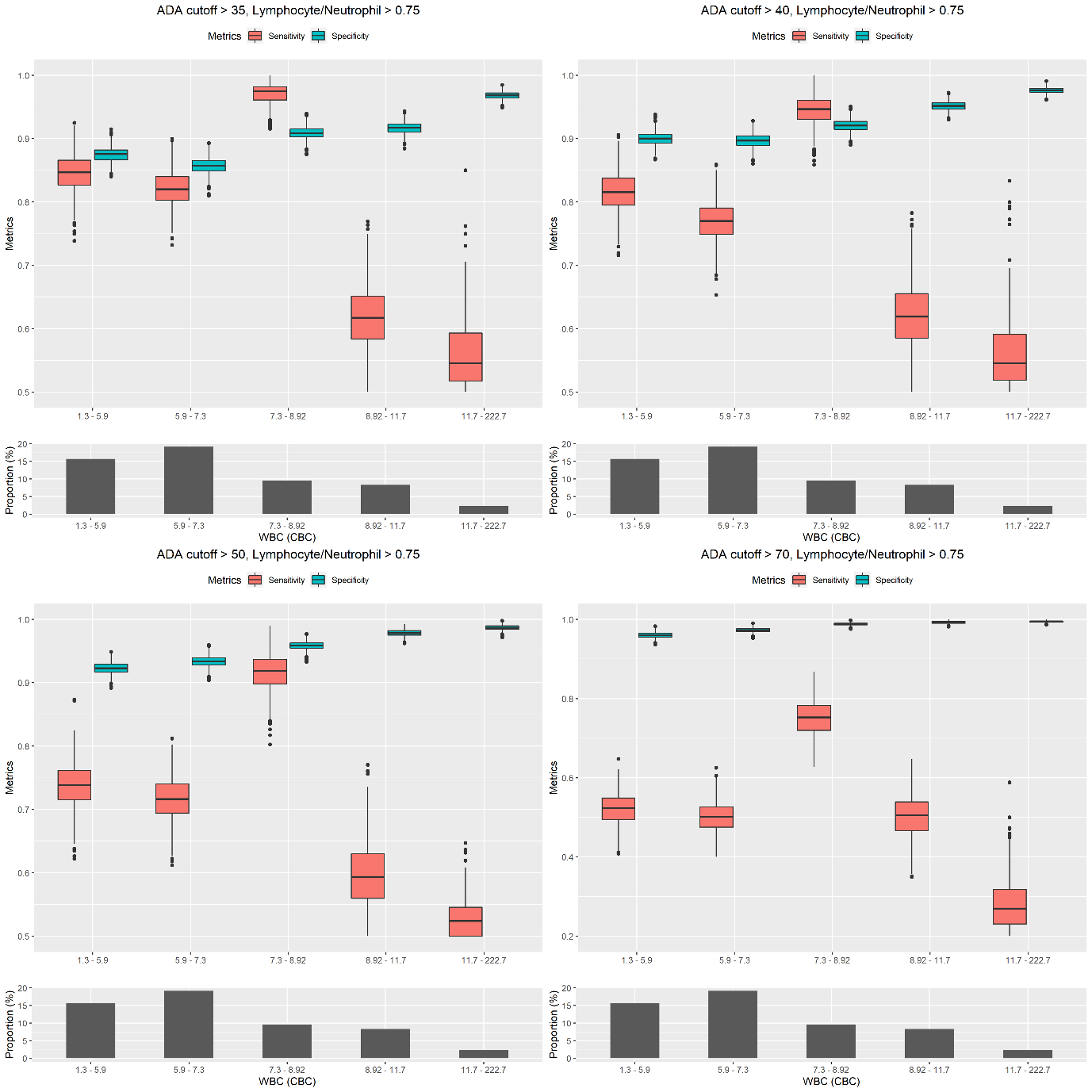


Patients were categorised into groups according to their WBC by five quantile ages. The sensitivity and specificity of various criteria in the simulated random datasets are described in the box plot. The proportion of tuberculosis in each quartile group is presented as a bar plot.

Supplement Figure 2. Simulation of diagnostic accuracy of various L/N ratios according to WBC group.


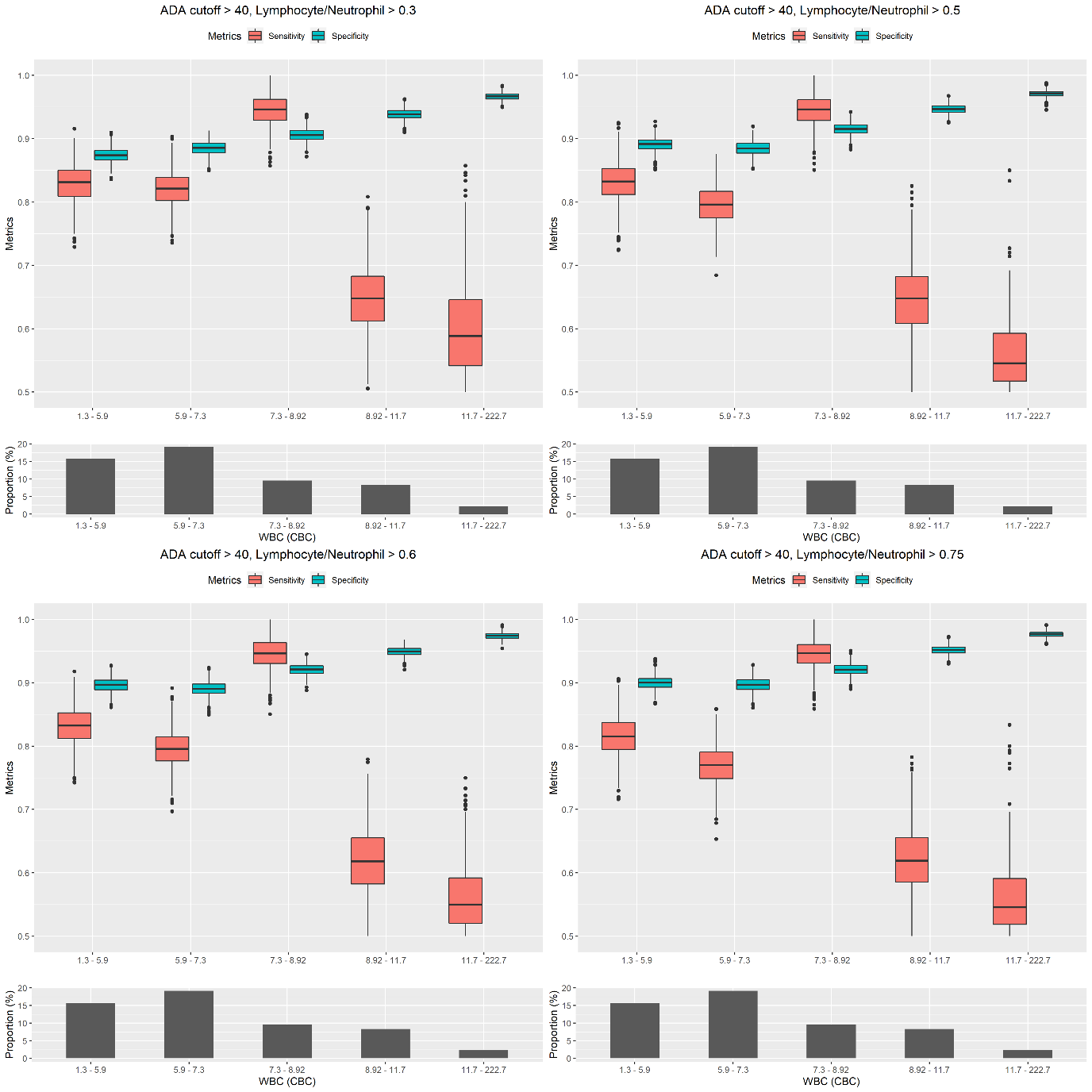


Supplement Figure 3. Diagnostic metrics according to various cut-off criteria for tuberculous effusion by CRP group.


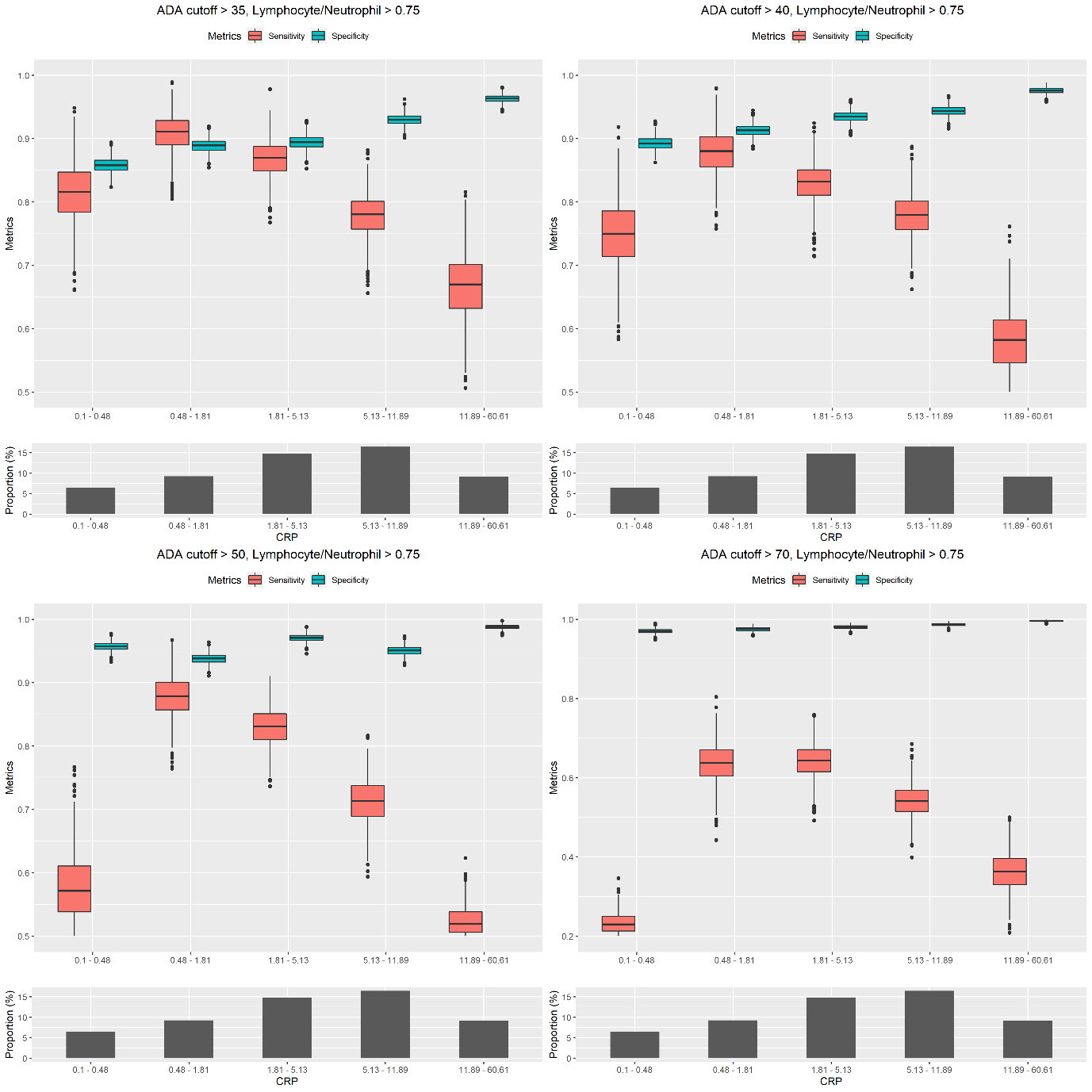


Patients were categorized into groups according to their CRP by five quantile ages. The sensitivity and specificity of various criteria in the simulated random datasets are described in the box plot. The proportion of tuberculosis in each quartile group is presented as a bar plot.

Supplement Figure 4. Simulation of diagnostic accuracy of various L/N ratios according to CRP group.


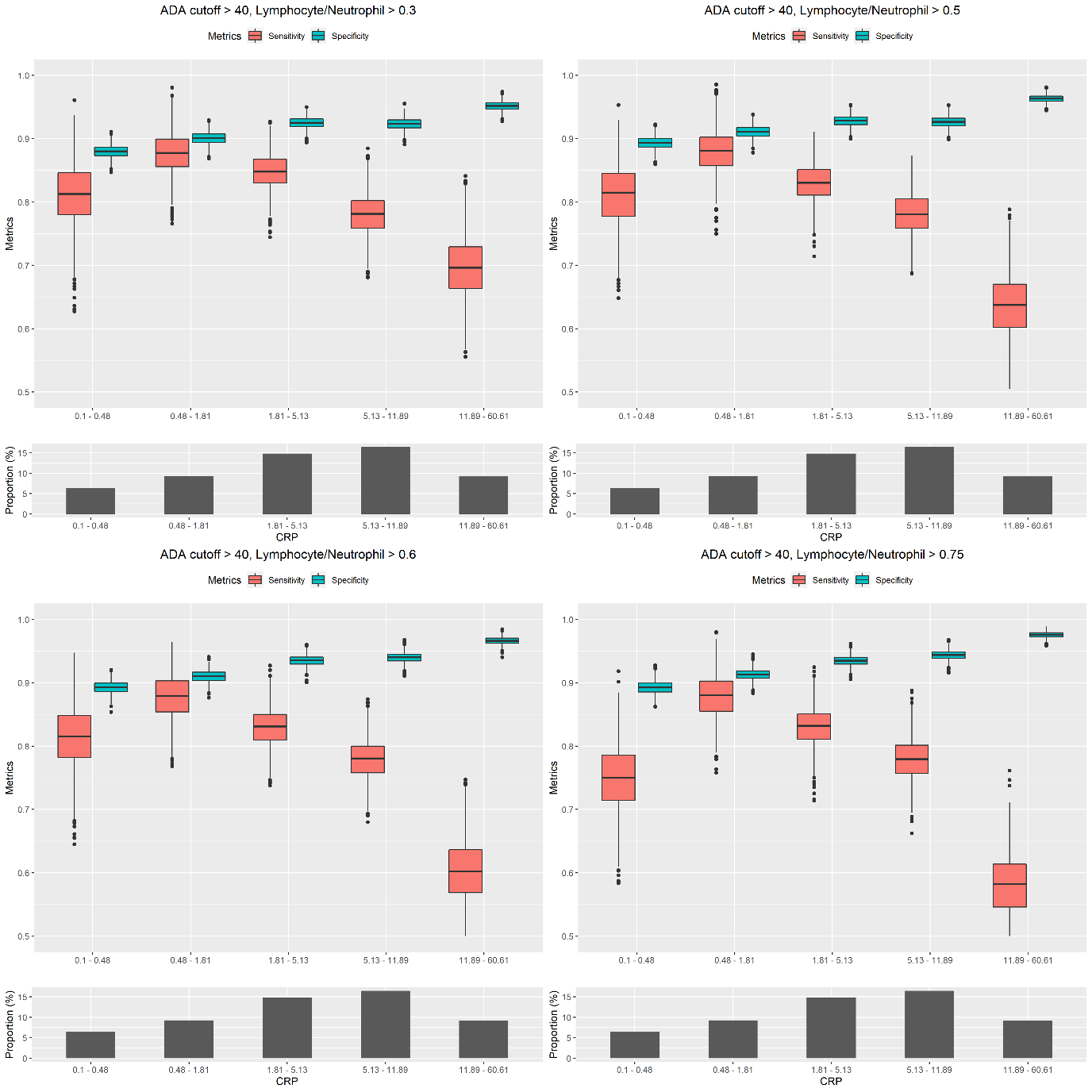


Supplement Figure 5. The proportion of aetiologies according to quantile group.


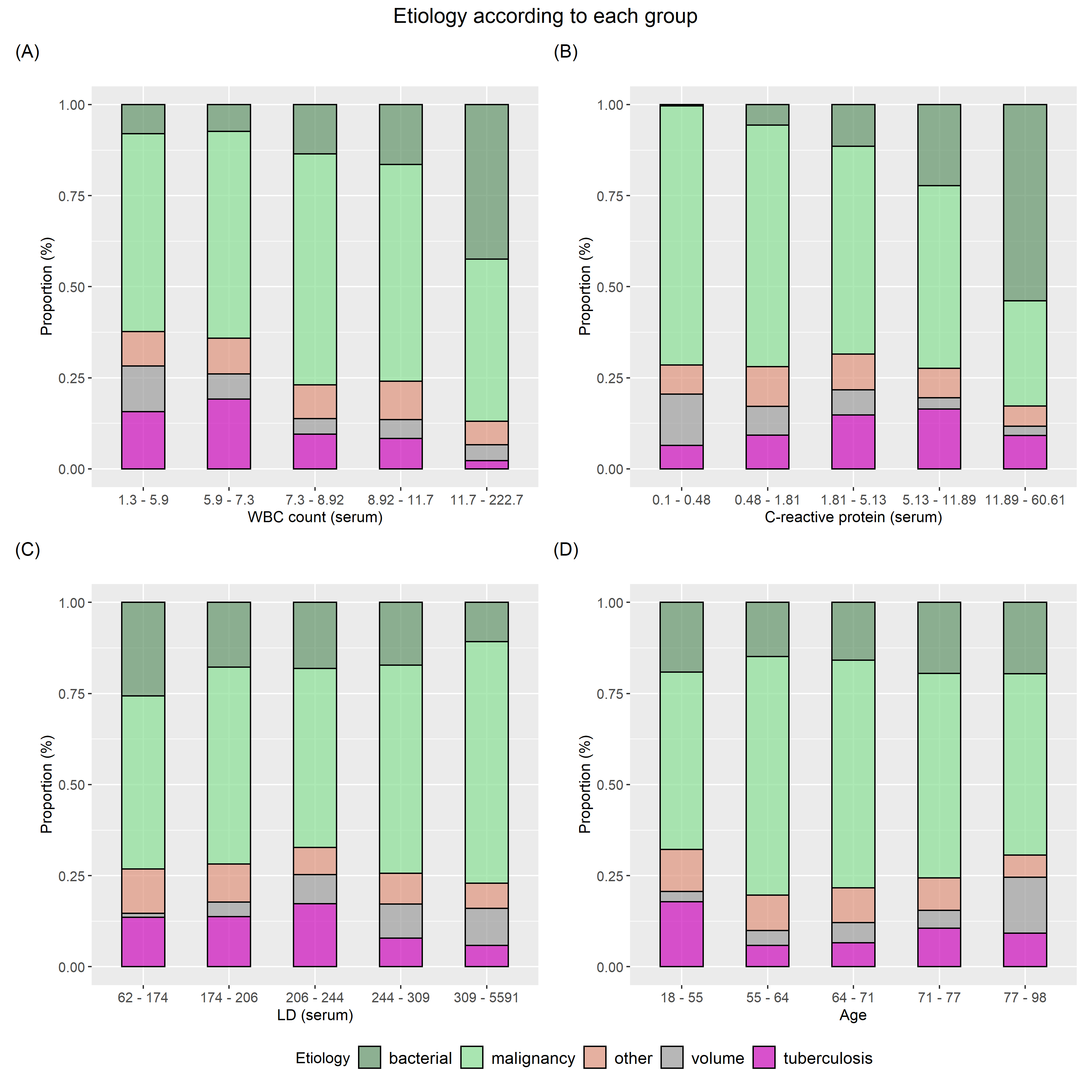


The aetiologies of pleural effusion according to each quantile group are depicted by stacked bar plots. (A) The proportion of aetiologies in the WBC quantile group. (B) The proportion of etiologies in the CRP quantile group. (C) The proportion of aetiologies in the LD quantile group. (D) The proportion of aetiologies in the age quantile group.

Supplement Figure 6. The association of ADA and age in tuberculous pleurisy


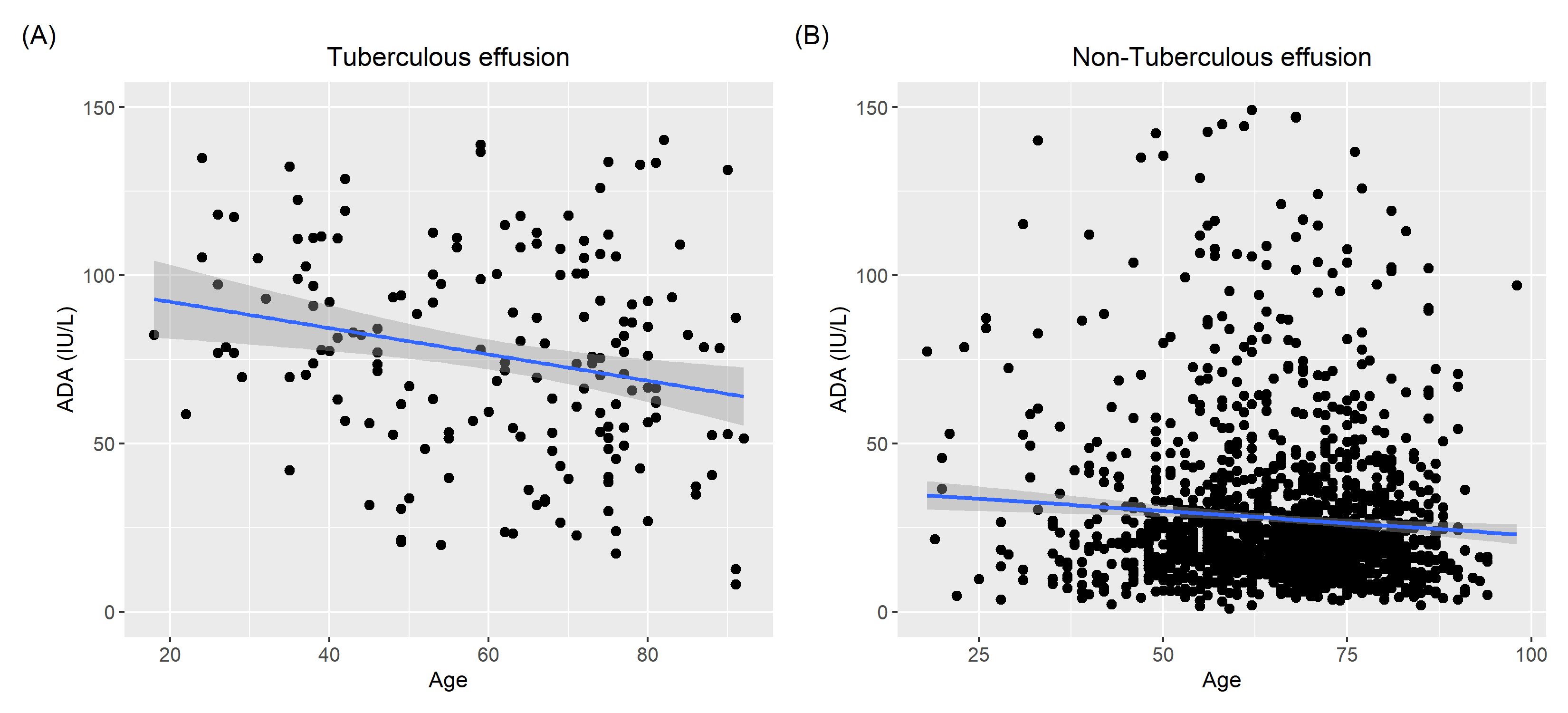


Age was negatively associated with ADA levels in tuberculous effusion; however, no significant association was observed in non-tuberculous effusion. The 1-year increase had lower ADA levels in tuberculous and non-tuberculous effusion at -0.449 and -0.147 (95% confidence interval tuberculous: -0.880 to -0.02; non-tuberculous: -0.439 to 0.144).

Supplement Figure 7. Diagnostic metrics according to various cut-off criteria for tuberculous effusion by age group.


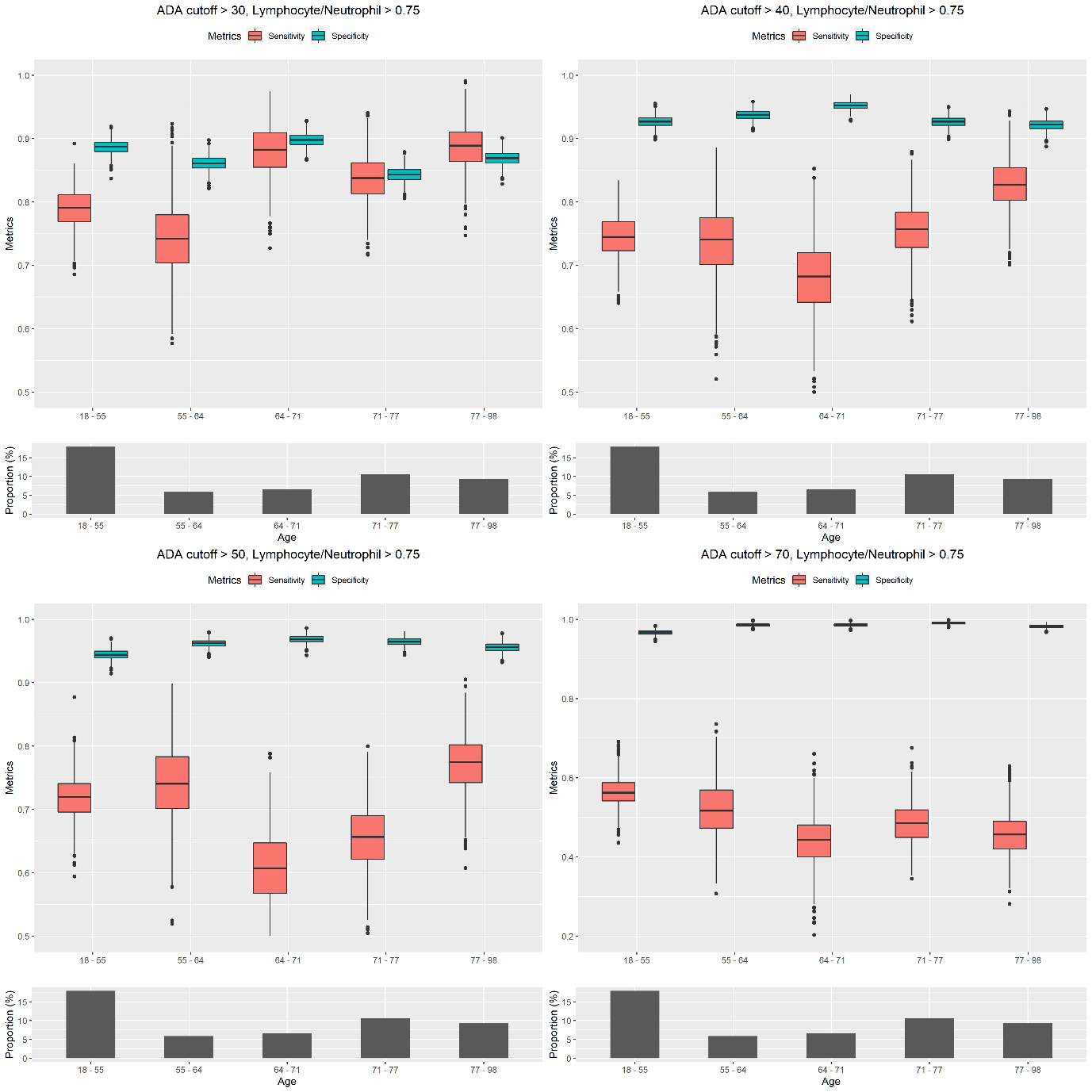


Patients were categorised according to age by five quantile ages. The sensitivity and specificity of various criteria in the simulated random datasets are described in the box plot. The proportion of tuberculosis in each quartile group is presented as a bar plot.

Supplement Figure 8. Simulation of diagnostic accuracy of various L/N ratios cut-offs according to age group.


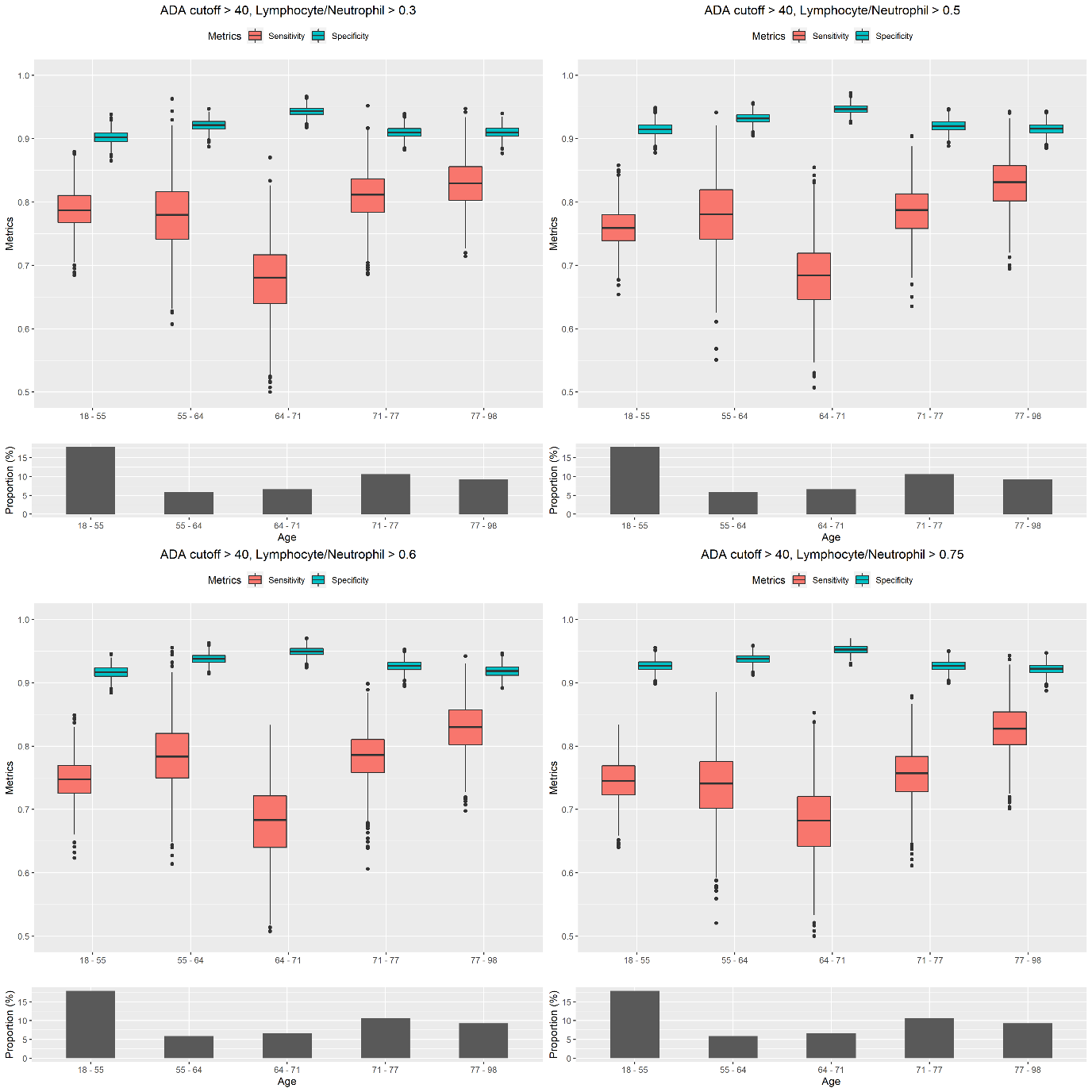


**Supplementary table 1.** The results of bacterial culture in pleural fluid

| **Pathogen** | **Number of Patients (n=33)** |
| --- | --- |
| Staphylococcus epidermidis | 8 |
| Staphylococcus hominis | 3 |
| Candida albicans | 2 |
| Klebsiella pneumonia | 2 |
| Acinetobacter baumannii | 1 |
| Bacillus spp. | 1 |
| Candida glabrata | 1 |
| Candida parapsilosis | 1 |
| Candida tropicalis | 1 |
| Corynebacterium spp. | 1 |
| Enterococcus faecalis | 1 |
| Enterococcus faecium | 1 |
| Escherichia coli | 1 |
| Haemophilus parainfluenzae | 1 |
| Peudomonas aeruginosa | 1 |
| Rothia spp. | 1 |
| Staphylococcus aureus | 1 |
| Staphylococcus haemolyticus | 1 |
| Streptococcus agalactiae, group B | 1 |
| Streptococcus anginosus | 1 |
| Streptococcus mitis | 1 |
| Streptococcus pneumoniae | 1 |

**Supplementary table 2.** Study markers in culture-positive and neutrophil-dominant tuberculous pleural effusion

|  | **Culture-positive tuberculous effusion**  **(n = 19)** | **Neutrophil-dominant tuberculous effusion**  **(n = 23)** | **Total tuberculous effusion**  **(n = 229)** |
| --- | --- | --- | --- |
| ADA > 50 U/L | 14 (73.7%) | 19 (82.6%) | 190 (83.0%) |
| L/N ratio > 0.75 | 10 (52.6%) | N/A | 203 (88.6%) |
| ADA > 50 U/L  and L/N ratio > 0.75 | 6 (31.6%) | N/A | 169 (73.8%) |
